# Refractory Classical Hodgkin Lymphoma Presenting with Atypical Cutaneous Involvement and Diagnosis of ZZ Phenotype Alpha-1 Antitrypsin Deficiency

**DOI:** 10.1155/2014/642868

**Published:** 2014-05-13

**Authors:** Mohamad Khawandanah, Teresa Kraus, Mohamad Cherry

**Affiliations:** ^1^Hematology-Oncology Section, Department of Medicine, Stephenson Cancer Center, The University of Oklahoma Health Sciences Center, 800 NE 10th Street, Oklahoma City, OK 73104, USA; ^2^Department of Pathology, The University of Oklahoma Health Sciences Center, 940 Stanton L. Young Boulevard, BMSB 451, Oklahoma City, OK 73104, USA

## Abstract

Cutaneous Hodgkin lymphoma is a rare condition. Specific neoplastic involvement can be primary (confined to the skin) or secondary to systemic involvement (metastatic). Cutaneous involvement by HL usually occurs late in the course and is associated with poor prognosis; however in some cases it can exhibit indolent behavior. Skin involvement with nonspecific cutaneous findings may represent a paraneoplastic syndrome. We describe a case of 46-year-old white male patient presented with rash and lymphadenopathy which led to the diagnosis of stage IVE mixed cellularity classical Hodgkin lymphoma with skin involvement. His disease was refractory to multiple lines of chemotherapy including (1) AVD (doxorubicin/bleomycin/dacarbazine), (2) brentuximab, and (3) bendamustine, he later achieved complete remission with (4) GCD (gemcitabine/carboplatin/dexamethasone) salvage regimen. Bleomycin was not given secondary to poor pulmonary function tests. His treatment was complicated after AVD with multiple pneumothoraces which unmasked the diagnosis of ZZ phenotype alpha-1 antitrypsin (ATT) deficiency. Simultaneous existence of Hodgkin lymphoma and ATT is rarely reported.

## 1. Introduction

Hodgkin lymphoma (HL) is a highly curable malignancy and represents around 10% of all lymphomas and approximately 0.6% of all cancers diagnosed annually [1]. It originates from germinal center or postgerminal center B cells, and a unique hallmark is in presence of Reed-Sternberg cells. Hodgkin lymphoma is classified as either classical or nodular lymphocyte predominant. According to the 2008 WHO classification, classical HL is subdivided into nodular sclerosis (70%), mixed cellularity (20 to 25%), lymphocyte rich (5%), and lymphocyte depleted (less than 1%) [2]. Management is broadly similar in all subtypes.

Hodgkin lymphoma typically presents as painless lymphadenopathy, which is frequently cervical, supraclavicular, and/or mediastinal. Cutaneous Hodgkin lymphoma is a rare condition that usually occurs late in the course of disease. Presentation with skin involvement has been reported in few cases [3]; however in primary cutaneous disease or skin only disease the incidence is exceedingly rare [4]. The incidence is variable from 0.5 to 3% [5, 6].

On the other hand, alpha-1 antitrypsin (AAT) deficiency is relatively common but underrecognized with less than 10% of the estimated 100,000 Americans with AAT who were diagnosed [7]. Neutrophil elastase (NE) and ATT are a pair of protease and protease inhibitor counterparts that play a role in ATT deficiency pathogenesis [8]; ATT is serine protease inhibitor and A1 (SERPINA1) is an important serine protease inhibitor in humans. One of the main physiological roles of AAT is to inhibit NE released from triggered neutrophils, with an additional lesser role in defense against damage inflicted by other serine proteases, such as cathepsin G and proteinase 3. Imbalance between NE and AAT can lead to tissue damage and subsequently may lead to carcinogenesis and tumor progression.

Deficiency of ATT has been described in association with increased risk of liver cancer, bladder cancer, gall bladder cancer, malignant lymphoma, and lung cancer. Elevated levels of NE may promote the development, invasion, and metastasis of many cancers [17]. Association of Hodgkin lymphoma and alpha-1 antitrypsin deficiency is rare, and the incidence of ATT deficiency in HL patients is around 5% [14].

## 2. Methods

We searched Medline and PubMed for articles published under the term “cutaneous Hodgkin lymphoma” and the related terms “skin involvement,” “alpha1 anti trypsin deficiency,” “alpha-1 antitrypsin deficiency phenotypes,” and “relapsed Hodgkin lymphoma” or “refractory Hodgkin lymphoma.” We did not restrict references by language of publication, and references from relevant articles were also searched.

## 3. Case Presentation

**Figure 1:**
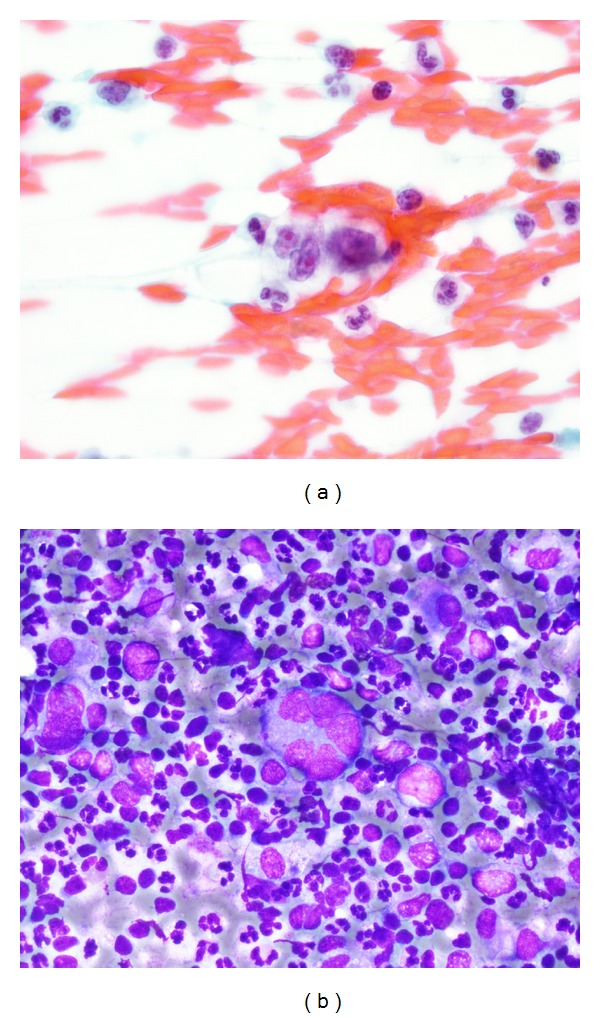
Pathology microscopy of FNA of the chest wall lesion on presentation: (a) 100x magnification, Papanicolaou stain: Reed-Sternberg cell in a mixed inflammatory background, and (b) 60x magnification, Diff Quik (modified Giemsa) stain: Reed-Sternberg cell in a mixed inflammatory background consisting of neutrophils, histiocytes, and small lymphocytes.

A 46-year-old white male patient presented to our facility in July 2011 with complaints of painless lumps in his right side of the neck, right axilla, and left groin that appeared 3 months prior to presentation. He also noted a light red rash involving the right side of the neck extending to the right upper chest appeared simultaneously. He complained of effortless weight loss and fatigue. He denied any febrile illness or night sweats. He had a history of obstructive lung disease and GERD and underwent appendectomy as a child. He had a long standing history of smoking. There was no significant family history or known drug allergies. His physical exam was remarkable for cervical, right axillary, and left inguinal lymphadenopathy, in addition to a homogenous erythematous rash involving the upper chest and left neck. His initial laboratory work-up showed a hemoglobin of 12.3 g/dL, WBC 33,800/mm^3^, with a differential of 88% segmented neutrophils, 1% band, 5% lymphocytes, 4% monocytes, and 2% eosinophils, and a platelet count of 456,000/mm3. LDH was mildly elevated at 212 U/L. Liver and kidney chemistry profiles were unremarkable. HIV and hepatitis serology tests were negative. A PET/CT scan showed extensive hypermetabolic anterior chest wall uptake with peak SUV 15.1, in addition to uptake in bilateral cervical, mediastinal, paraesophageal, precarinal, right internal mammary, left subpectoral, and right posterior paraspinal lymph nodes (LN). There was no FDG-avid uptake below the diaphragm. A large upper lung lobe bulla and smaller bullae and blebs in the left lower lobe were noted incidentally. The patient was admitted to the hospital to expedite work-up and obtain tissue diagnosis. He underwent fine needle aspiration (FNA) of the rash on the chest wall, which revealed large atypical cells consistent with Reed-Sternberg cells in a mixed inflammatory background, suspicious of HL (see Figure 1). FNA of right axillary also showed findings suspicious of HL (not shown).

**Figure 2:**
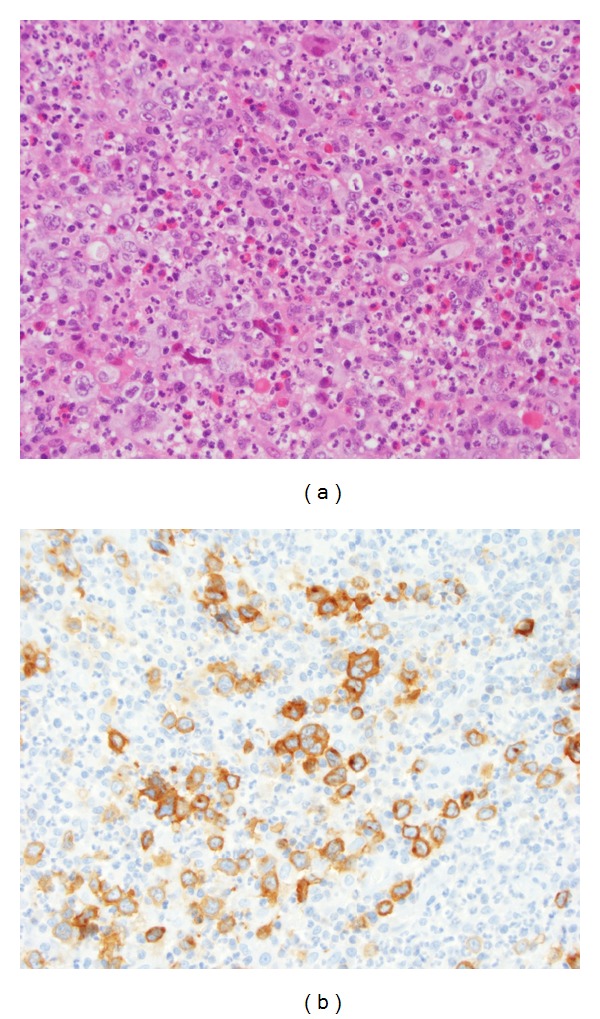
Pathology microscopy of excisional biopsy of a right cervical lymph node (a) showed effacement of the lymph node architecture by a mixed infiltrate of neutrophils, eosinophils, histiocytes, and small lymphocytes, with scattered Reed-Sternberg cells (H&E stain 40x). (b) Immunohistochemical staining for CD30 highlighted the Reed-Sternberg cells (40x).

Subsequently patient underwent open excisional biopsy of right cervical and left inguinal LN. His inguinal LN was benign with reactive changes, while the cervical biopsy was diagnostic for classical HL, mixed cellularity (see Figure 2). A bone marrow exam was normal. His final staging was IIE or considering the disease multifocal with his skin disease his stage would be IVE.

**Figure 3:**
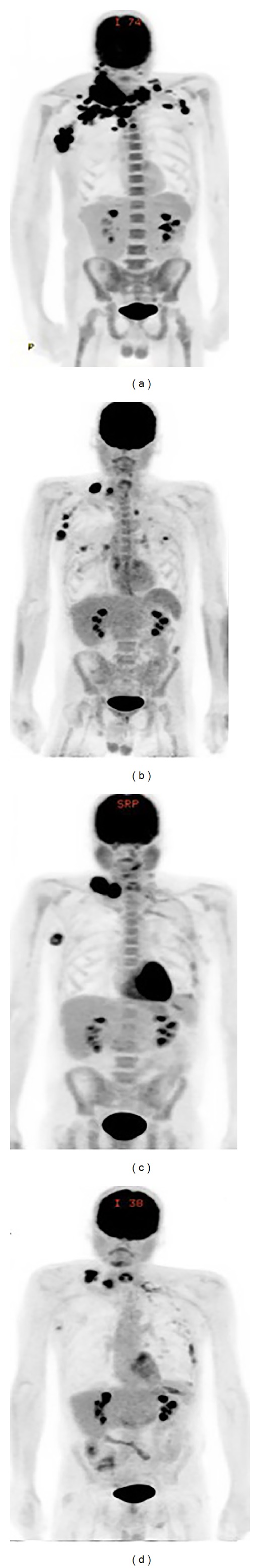
(a) PET scan at presentation, (b) PET scan done after 6 cycles of AVD, (c) PET scan after 8 cycles of AVD, and (d) PET scan after 2 cycles of brentuximab.

At this point our decision was to treat the patient with standard ABVD (doxorubicin, bleomycin, vinblastine, and dacarbazine), which is the standard of care, but unfortunately his pulmonary function test results were poor. His DLCO was 35%, while his FEV1/FVC was 32% of predicted value. We decided to start treatment with AVD without bleomycin (Table 1). Cycle 1 was complicated with dental abscess and interruption of treatment. After completion of 6 cycles he achieved partial response. The patient started to complain of chest pain, dyspnea, and anxiety and presented to our ER with tachycardia and hypoxia. A CT scan was obtained to rule out pulmonary embolism which again demonstrated bullae and severe emphysematous changes. He subsequently developed spontaneous pneumothoraces involving both sides of the lungs and required 4 chest tubes. His hospital course was protracted and required multiple talc pleurodesis procedures. This raised suspicion of alpha-1 antitrypsin deficiency as the cause of his pulmonary complications and led to more work-up. His AAT level was 30 mg/dL; repeat testing for verification showed level of 17 mg/dL (normal range is 90–200 mg/dL). The AAT phenotype was homozygous ZZ. Two months later, the patient was discharged from the hospital with a left side Heimlich flutter valve and home oxygen. He was requiring only 2 L/min of oxygen by nasal cannula. We treated him with an additional 2 cycles of AVD and his disease progressed. He developed left cervical flesh-like lesion on top of his lymphadenopathy. Biopsy was not feasible and patient declined bone marrow biopsy for restaging. Due to his multiple medical problems and poor performance status, we decided to treat him with IV brentuximab vedotin every 3 weeks as we believed he could not tolerate intensive salvage cytotoxic chemotherapy. He tolerated this biologic agent with no complications and his original rash improved significantly. Interim follow-up PET scan showed positive, but incomplete, response (see Figure 3). After 8 cycles, he developed bulky progression on PET scan evaluation, mainly in the right supraclavicular area, with SUV of 12, in addition to having stable disease in the right axilla. The patient was not considered for stem cell transplant secondary to his severe pulmonary disease.

Restaging FNA of his supraclavicular lesion confirmed the same diagnosis of HL, but his axillary lesion was negative for malignancy. The repeated bone marrow exam remained normal. A third line chemotherapy regimen consisting of IV bendamustine every 3 weeks started thereafter. He tolerated this regimen but progressed after 2 cycles. His rash recurred again on the same area on the chest covering the supraclavicular LN with ulcerated nodule and flesh color. At that point we referred the patient for radiation therapy, but this was not feasible as he could not lay flat secondary to severe orthopnea.

**Figure 4:**
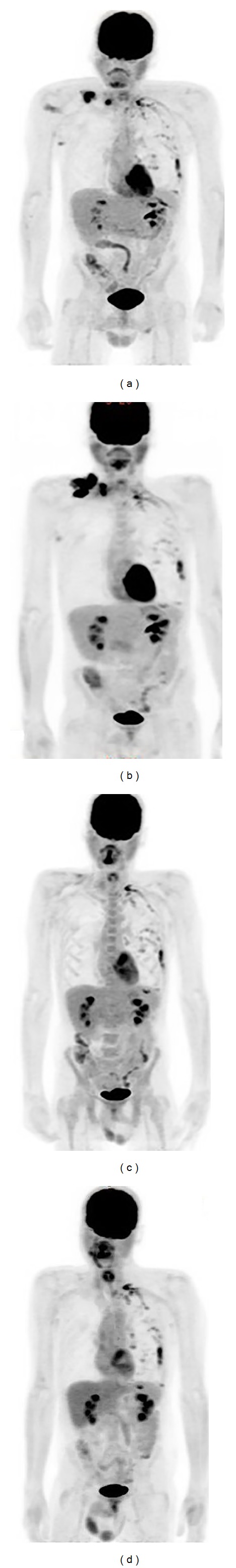
(a) PET scan during brentuximab therapy, (b) PET scan after 2 cycles of bendamustine, (c) PET scan after 2 cycles of GCD, and (d) PET scan at conclusion of treatment with GCD. Pleurodesis inflammatory changes remained on imaging.

The patient requested to be treated; however options were limited for clinical trial enrollment in his case, so we treated him with fourth line salvage chemotherapy regimen consisting of gemcitabine/carboplatin/dexamethasone (GCD) with growth factor support. Therapy was complicated with interruptions and dose reductions secondary to hematologic toxicities. Postcycle 2 PET scan showed near complete remission without clinically palpable lymph nodes. He remained oxygen dependent and his rash resolved completely. The patient noticed increased dyspnea after cycle 3 within days after chemotherapy, and he requested discontinuation of therapy after cycle 5. Posttreatment PET scan was compatible with complete metabolic remission (see Figure 4).

Currently, the patient is being treated with intravenous augmentation via the infusion of pooled human alpha-1 antiprotease. He is doing well 8 months after stopping chemotherapy, with no evidence of lymphoma recurrence.

## 4. Discussion

Our case is unique, as rash is a very uncommon initial presentation of Hodgkin lymphoma. Furthermore, the association between lymphoma and alpha-1 antitrypsin deficiency is scarcely described in the literature.

Cutaneous Hodgkin lymphoma was first described by the German physician Grosz in 1906 [18]. This rare condition is thought to have decreasing incidence in recent decades, owing to the improved treatment modalities of patients with Hodgkin lymphoma. In addition to rarity, skin involvement carries an ominous prognosis but can be indolent if systemic disease was controlled properly [19]. Cerroni et al. found that histology of cutaneous specific manifestations of Hodgkin lymphoma correlates with that of the nodal counterpart in most cases [20].

Cutaneous involvement can range from simple erythematous rash to spectrum of papular, nodular, nodular-ulcerative, plaque-like, and infiltrative lesions [6, 21, 22] (see Table 2 for ulcerative lesion types). Other nonspecific skin manifestations in patients with Hodgkin lymphoma include Addisonian pigmentation, pruritus, prurigo eruption, acquired ichthyosis, erythroderma, alopecia, and recurrent herpes zoster infection [23].

Callea et al. studied ATT phenotypes of 228 patients diagnosed with lymphoma and 250 healthy controls and concluded that there was an increased incidence of abnormal phenotypes in lymphoma patients, with no prevalence for a special lymphoma type (Table 3). In the same report, 7 patients were described with Hodgkin lymphoma and MZ phenotype in addition to 1 patient with unknown status [14].

Although there is a reported association between AAT polymorphism and different types of cancer, this association with hematological malignancies is unknown [24]. Topic et al. described a study of identification of ATT phenotypes in 151 patients with hematologic malignancies and 272 healthy controls. There was no difference in the frequency of deficient AAT alleles (Pi Z and Pi S) between patients and controls, but there was higher frequency of PiM1M1 homozygote and PiM1 allele in patients with hematologic malignancies, suggesting that even AAT polymorphisms with functionally normal phenotypes may be associated with an increased risk of hematologic disease.

## 5. Conclusions

We present a unique case of skin involvement with Hodgkin lymphoma that followed the systemic pattern of the disease in terms of response to chemotherapy. Treatment of our patient's refractory lymphoma was challenging due to his poor performance status and pulmonary complications. Despite the diagnosis of alpha-1 antitrypsin deficiency, he tolerated multiple lines of chemotherapy. Simultaneous existence of both disorders is rarely reported.

Regardless of primary or secondary lymphoma origin of skin lesions, treatment should be directed systematically with conventional chemotherapy. Judicious chemotherapy selection is important in such refractory cases.

## Figures and Tables

**Table 1:** List of chemotherapy agents utilized in our case.

Regimen	Description	Number of cycles given	Reference
AVD without bleomycin	Doxorubicin 25 mg/m^2^ IV on days 1 and 15 Vinblastine 6 mg/m^2^ IV on days 1 and 15 Dacarbazine 375 mg/m^2^ IV on days 1 and 15	8	[9]

Brentuximab vedotin	Brentuximab 1.8 mg/kg IV on day 1	8	[10]

Bendamustine	Bendamustine 120 mg/m^2^/day IV on days 1 and 2	2	[11]

GCD	Gemcitabine 1000 mg/m^2^/day IV on days 1 and 2 Carboplatin AUC 5 IV on day 1 Dexamethasone 40 mg po on days 1, 2, 3, and 4	5	[12]

**Table 2:** Types of ulcerative lesions in cutaneous Hodgkin lymphoma.

Types of ulcerative lesions	Description
Grosz-Hirschfield	Ulceration develops on nodule
Coleman-Anderson	Ulceration develops in skin directly infiltrated from underlying lymph node or other tissues
Dossekker-Kren-Saalfeld	Occurs when a primary cutaneous Hodgkin lymphoma ulcerates

**Table 3:** Correlation between ATT phenotype and emphysema, lymphoma, and lung cancer.

Phenotype	Risk of emphysema [13]	Incidence of lymphoma [14]	Risk of lung cancer [15, 16]
MM (normal)	No increase	Baseline risk	Baseline risk
MZ	Possible mild increase	5.8%	Hypothetical increased risk
SS	No increase	0.4%	
SZ	20 to 50%	n/a	
ZZ	80 to 100%	n/a	
Null	100% by age 30	n/a	
